# Issues contributing to non-attendance for chronic illness care for hepatitis C

**Published:** 2012-09-04

**Authors:** Gail Butt, Liza McGuinness, Adrienne Peltonen, Sandra Mitchell

**Affiliations:** Canada British Columbia Centre for Disease Control, University of British Columbia, Canada; University of British Columbia, Canada; University of British Columbia, Canada; British Columbia Centre for Disease Control, Canada

**Keywords:** chronic illness, hepatitis C, non-attendance, qualitative research

## Abstract

**Purpose:**

To identify and describe the personal and systems factors related to not seeking, delaying or deferring chronic illness care for hepatitis C.

**Theory:**

A multi-level ecological theory guided the analysis, organization and interpretation of findings.

**Methods:**

This qualitative study, nested within a larger exploration and intervention study, used an interpretive descriptive method. Data were collected through a demographic questionnaire and open-ended individual and focus group interviews which were transcribed, coded and subjected to thematic analysis.

**Results and conclusions:**

Data were obtained from 4 focus groups, attended by 29 health and social service providers, and 55 interviews with affected individuals (55% male) from five Canadian provinces. Issues contributing to non-attendance were identified at multiple levels: personal, interpersonal, provider-system and structural. Key themes contributing to non-attendance were previous negative experience, provider and/or client disease knowledge and communication, stigma, restrictive policies, treatment eligibility criteria, personal priorities, poverty and unstable lifestyles.

This is the first national study focused on chronic hepatitis C that illuminates the reasons for non-attendance at care from both the user and provider perspectives. The next step is to work with those affected and providers to develop client and provider resources that will address the issues identified and improve service quality and uptake.

Powerpoint presentation available at http://www.integratedcare.org at congresses – San Marino – programme.

